# Characterization of T-cell receptor loci and expressed repertoire reveals a capacity for robust T-cell response in Atlantic cod (*Gadus morhua*)

**DOI:** 10.1038/s41598-026-45018-x

**Published:** 2026-03-22

**Authors:** Ádám Györkei, Finn-Eirik Johansen, Shuo-Wang Qiao

**Affiliations:** 1 Department of Immunology, Institute of Clinical Medicine, University of Oslo, Oslo, Norway; 2 Section for Physiology and Cell Biology, Department of Biosciences, University of Oslo, Oslo, Norway

**Keywords:** T-cell receptor, T-cell receptor repertoire, Atlantic cod, Immune receptor genome, Computational biology and bioinformatics, Genetics, Immunology

## Abstract

The Atlantic cod presents a peculiar case in fish immunology, due to the loss of MHCII and CD4 genes. Despite the absent antibody response to T cell-dependent antigens, it is a prolific species. Characterization of T-cell receptor loci and the expressed repertoire is necessary to further our understanding of how the Atlantic cod can mount an effective immune response and to characterize its immune system on the molecular level. A comprehensive annotation of the Atlantic cod genome (gadmor3.0) revealed all four chains of T-cell receptors, residing on chromosomes 5 and 8. Moderate germline diversity was observed with signs of recent expansion in the triplicate TRB locus. Sequencing of the expressed repertoire showed a surprisingly high repertoire diversity for TRA, TRB and TRD chains. Only small fractions of the repertoires were public with further untapped diversity available through differential gene usage especially for TRA and TRD chains. Our study revealed that despite its modest genomic T-cell receptor sequence diversity, the Atlantic cod maintains a diverse, mostly private expressed repertoire. This work provides a baseline for comparative studies and investigations of TR repertoire changes during vaccination and infection experiments.

**Supplementary Information:**

The online version contains supplementary material available at 10.1038/s41598-026-45018-x.

## Introduction

T cells are central components of the adaptive immune system across jawed vertebrates, including mammals and fish^1^. They play diverse roles in both cellular and humoral immune responses and contribute to the formation of immunological memory. Antigen recognition by T cells is mediated through their surface-bound T-cell receptors (TRs), which are heterodimeric molecules composed of either alpha and beta or gamma and delta chains. Alpha-beta T cells recognize peptide antigens presented by major histocompatibility complex (MHC) class I and class II molecules, whereas gamma-delta TRs have the ability to directly bind antigens in a manner more similar to immunoglobulins^2^. They go through different maturation processes, with alpha-beta T cells requiring positive selection on MHC, whereas some populations of gamma-delta cells can survive without it^3^. The distribution of these T cell subsets also varies, with alpha-beta T cells predominating in blood, while gamma-delta T cells are more abundant at mucosal surfaces such as the gut. Notably, interspecies variation in gamma-delta T cell prevalence is considerable; in some species, mostly ruminants, gamma-delta T cells constitute nearly 50% of circulating lymphocytes^4^; whereas, other species, including the pipefish, the blunt-snouted clingfish^5^, and squamates^6^, completely lack the gamma and delta loci. T-cell receptor diversity is generated through somatic V(D)J recombination, where beta and delta chains undergo rearrangement of variable (V), diversity (D) and joining (J) segments, while alpha and gamma chains recombine only V and J segments. This initial diversity is further expanded by deletions and non-templated nucleotide insertions at the recombination junctions, followed by the combinatorial pairing of alpha and beta or gamma and delta chains, collectively shaping the antigen-binding repertoire. The germline gene pool and its organization strongly influence the final TR repertoire size; however, genomic loci for TR genes have only been characterized in a limited number of fish species.

The T-cell receptor (TR) loci exhibit significant structural diversity across teleost species, influencing immune function and antigen recognition. TR alpha/delta locus maintains a conserved translocon organization between fish and mammals, but its gene orientation differs—V genes are in inverted orientation relative to D, J and C (constant) genes, with TRD genes preceding TRA genes in fish. This inversion allows secondary recombination events, potentially expanding receptor diversity. Among studied teleosts, Atlantic salmon has the most diverse TR alpha/delta germline repertoire^7^(292 V genes, 122 J genes), though more than half are pseudogenes, leaving 128 functional V genes. Other species exhibit substantial variation; rainbow trout strains show marked differences in V and J gene counts^8^, zebrafish retain a high proportion of functional genes^9^(141/149 V genes), and channel catfish possess the largest described TR alpha/delta locus (1.285 Mb)^10^. In contrast, pufferfish have a significantly reduced germline repertoire^11^, with only 13 V genes.

The TR beta locus follows a translocon structure but displays remarkable variation in germline diversity, driven by whole-translocon duplications in zebrafish^12^ and large yellow croaker^13^, and whole-genome duplications in salmonids^14^. The Arlee strain of rainbow trout is especially notable, since it contains two complete TRB loci in divergent orientation on chromosome 25 and another complete locus on chromosome 19^15^. Zebrafish possess two alternative C genes, one of which facilitates direct VJ recombination, bypassing the D segment. Channel catfish exhibit the highest germline TRBV diversity^10^(112 V genes, 29 subgroups), while Atlantic salmon has two distinct TRB loci on chromosomes 1 and 9, each with different number of V, D and J genes and V gene usage^14^, providing a potential for functional divergence.

The TR gamma locus shows the greatest structural variability, but lowest overall genomic diversity, with species following either a mini-cluster arrangement^16^(“mouse-like”) or a translocon organization^17^(“human-like”). Channel catfish^10^ exhibit a mini-clustered TR gamma genes, while zebrafish retain a single translocon^10^. Atlantic salmon possesses two distinct TR gamma loci^18^, one arranged in mini-clusters, the other forming a single V-J-C unit, with differences in C-region connecting peptides, suggesting functional divergence beyond simple gene duplication. These findings underscore the evolutionary flexibility of TR genes, with species-specific adaptations shaping immune strategies in response to different pressures. The number of germline genes for teleost species with genomic annotation is presented in Table 1.

**Table 1:** Number of TR gene segments in teleostei species. The number of functional genes are shown in brackets.

	alpha/delta						beta				gamma			Reference
	TRADV	TRAJ	TRAC	TRDD	TRDJ	TRDC	V	D	J	C	V	J	C	
**Salmon**	292 (128)	122 (113)	1	3	1	1	119 (100)	4	36	4	11	6	6	Yazawa (2008a), Yazawa (2008b)
	239 (104)	128	1	3	1	1								Grimholt (2022), Yazawa (2008b)
**Rainbow trout (Swanson)**	57 (25)	66	1	3	1	1	188 (160)	3	24	3	---	---	---	Edholm (2021)
**Rainbow trout (Arlee)**	164 (93)	81	1	3	1	1	188 (160)	3	24	3	---	---	---	Boudinot (2023)
**Zebrafish**	149 (141)	111	1	6	2	1	51	1	27	2	7	7	1	Seeyle (2016), Meeker (2010)
**Channel catfish**	140 (128)	125	1	4	1	1	112 (106)	1	31(29)	2	15 (13)	10 (8)	4	Crider (2021)
**Pufferfish**	13	12	1	4	2	1	---	---	---	---	---	---	---	Fischer (2002)
**Large yellow croaker**	---	---	---	---	---	---	31	2	13	2	---	---	---	Shi (2025)
**Atlantic cod**	52	21	1	1	1	1	34	3	23	3	7	7	6	This study

The Atlantic cod (*Gadus morhua*) represents a special case in fish immunology, distinguished by an unconventional adaptive immune system. Early studies reported weak antibody responses leading to speculation of compromised MHC class II function^19^, a hypothesis later confirmed by genomic analysis^20^. Further investigations revealed the absence of a functional CD4, suggesting a severely impaired T cell-dependent antibody response and a potentially deficient immunological memory, the existence of which remains unconfirmed. Given the weakened T cell-dependent humoral immunity in cod, its cellular immune response may exhibit distinct adaptations. These could manifest at the genomic level, through variations in TR gene repertoire and germline diversity, or at the expressed repertoire level, with differences in TR sequence diversity among circulating T cells. To date, research on cod T-cell receptors has been limited to partial characterizations of TR alpha and beta, with only a small number of chains have been sequenced due to technological constraints at the time^21^. These analyses identified four alpha and four beta V gene subgroups, a markedly lower diversity than observed in other teleosts, but lacked a comprehensive genomic assessment. The TR gamma and TR delta loci remained unannotated, leaving significant gaps in understanding how the Atlantic cods atypical immune architecture manifests on the T-cell receptor level.

In this study, we completed the genomic annotation of all TR loci in Atlantic cod, placing it among the few teleost species with fully characterized TR genomic sequences. By leveraging this genomic resource, we sought to analyze the expressed TR repertoire, providing insight into how germline diversity translates into functional repertoire diversity. Our goal is to test how the Atlantic cod T-cell receptor repertoire is shaped by its peculiar genetic background and provide a baseline for both future immunological challenge experiments and comparative analysis among fish species as more information on their repertoires become available.

## Methods

### Identification of TR genes in Gadmor3.0

Identification of T-cell receptor genes followed our previous approach of identifying B-cell receptor genes in the gadmor3.0 genome^22^. Briefly, regions of interest were identified by performing a sequence similarity search of a reference set of known V and C genes from IMGT^23^ with tBLASTn, with an e-value cutoff of 0.1. Genomic regions returning hits for both gene types simultaneously were extracted with at least 10 kb overhang from the first and last genes and analysed further. A more stringent similarity search with tBLASTn with a 0.00001 cutoff was conducted for V and C genes from IMGT, and a subsequent search using J genes with a 0.1 e-value cutoff in order to capture potential hits despite their shorter length. This outlined potential genes in the extracted genomic regions. Additional data was used from a whole transcriptome data from a prior study^24^, which was utilized to define intron-exon boundaries due to the several-fold drop in the number of mapped reads for the unspliced RNA compared to the spliced variant. 10 million reads were mapped in a splice-aware manner using STAR^25^ with default parameters, after trimming with Trimmomatic0.36^26^. A minimum quality score of 20 over a sliding window length of 4 was applied. Splice sites were validated manually and non-canonical splice sites were noted. Recombination signal sequences (RSS) were manually validated, after similarity search with canonical heptamer and nonamer sequence (CACAGTG and ACAAAAACC respectively) searches, then expanding the similarity search to single or double nucleotide changes repeated over multiple genes to identify non-canonical variants. D genes were identified by the two sets of RSS signals in opposite orientation, situated between V and J genes. Data from all sources were visualized in IGV^27^ and manually integrated. TR beta translocons were delineated by the 5’ most V gene and 3’ most C gene before the next set of V genes, and denoted translocon 1, 2 and 3. Gene nomenclature was used in accordance with the IMGT naming schemes, with the addition of the translocon number for TR beta genes as the first number after TRB, but before V, followed by a number that denoted the V gene subgroup and the last number that was unique for each V-gene. In case of TR gamma chains, V, J and C genes from the same mini-cluster always were assigned the same gene number. If a J or C gene was missing, the corresponding number was skipped, and duplicated genes received an additional suffix. Coding sequences were extracted and aligned with the MUSCLE algorithm^28^ and used in IMGT V-quest^29^ and Collier de Perles^30^ for the identification of complementarity-determining regions (CDRs) and framework regions. Phylogeny for V genes was created using MEGA11^31^ with the neighbour-joining method^32^ and 1,000 bootstrap replicates for confirming gene subgroup participation. The P-distance method quantified evolutionary distances, and ambiguous sites were removed in a pairwise fashion.

### 5’RACE sequencing of T-cell receptor repertoires

Atlantic cod were purchased from NOFIMA (Tromsø, Norway) and reared at the NIVA facility (Solbergstrand, Norway) as described earlier^33^. The experimental procedures were approved by the Norwegian authorities (FOTS ID 21758). Untreated cod were euthanised by cranial concussion, and sampling commenced immediately. Spleen was harvested from two cohorts of 6-month-old cod (weight 70–120 gram). The first cohort contained 4 fish with identifiers 0001, 0002, 0003 and 0016 and the second cohort contained fish 9019, 9020 and 9021. Harvested spleen was transported in RPMI media on ice and single-cell suspension was prepared within 6 hours of organ harvest. Two to ten million single spleen cells, corresponding approximately 15% of the total weight of the spleen, from each fish were homogenized in RLT lysis buffer (Qiagen) and kept frozen at −70 °C until RNA isolation. Given that around 20–25% of cells are T cells in spleen^33^, the estimated number of T cells in our sample was between 400,000 and 2.5 million. Total RNA was purified from the spleen cells using the RNeasy Plus Mini Kit (Qiagen) according to the manufacturer’s instructions. cDNA libraries of the T-cell receptor alpha, beta, gamma, and delta chains were prepared by 5’ RACE-PCR using template switch and nested C gene-specific primers located close to the V(D)J-junction. Technical replicates were created from the separately purified RNA of sample 9020 (indicated with a and b) for all chains. Only one replicate with the larger sampling depth was used in downstream analyses, as indicated. First strand cDNA was made from approximately 500 ng total RNA in the presence of a template-switch oligo (TSO) that enabled the incorporation of a universal priming site at the 3’-end of the first-strand cDNA corresponding to the 5’-end of mRNA. The first-strand synthesis reaction consisted of 1 µM oligo-dT primer (all primer sequences are found in Supplementary Table 2), 1 mM dNTP, 1x RT buffer, 2.5 mM DTT, 8 mM MgCl_2_, 1 M betaine (Merck), 0.8 U/µL Murine RNase Inhibitor (NEB), 1 µM TSO, and 8 U/µL Superscript II (Invitrogen) in a total volume of 40 µL. The reaction mix was incubated for 90 min at 42 °C, followed by an inactivation step of 72 °C for 15 min. In the first of total three semi-nested PCR reactions, 1.5 µL first strand cDNA was used as template where the TR alpha and beta chains were targeted in one PCR reaction and the gamma and delta chains in another PCR reaction. The first PCR reaction contained also 40 nM of STRTfwd_long primer, 200 nM each of the primers STRTfwd_short, TRAC_O and TRBC_O, or TRGC_O and TRDC_O, 200 µM dNTP, 1x Phusion high-fidelity buffer, 0.1 µL of Phusion polymerase (ThermoFisher) in a total reaction volume of 15 µL, and run with the cycling condition of denaturation 1 min at 98 °C; five cycles of (10s of 98 °C, 60 s of 72 °C); five cycles of (10s of 98 °C, 30 s of 70 °C, 40 s of 72 °C); eight cycles of (10s of 98 °C, 30 s of 68 °C, 40 s of 72 °C); and final extension of 4 min at 72 °C. In the following second PCR, each of the four TR chains were amplified in separate reactions each with 1 µL product from the first PCR reaction as template. In a total volume of 10 µL, the second PCR reactions contained 40 nM of STRTfwd_long primer, 200 nM each of the primers STRTfwd_short and TRAC_M, TRBC_M, TRGC_M or TRDC_M; 1x KAPA HiFi HotStart ReadyMix (Roche), and run with the cycling condition of denaturation of 2 min at 95 °C; 10 cycles of (15s of 98 °C, 30 s of 65 °C, 40 s of 72 °C); and final extension of 5 min at 72 °C. In the third PCR, 1 µL product from the second PCR reaction was used as template in each of the four third PCR reactions. In a total volume of 15 µL, each third PCR reaction contained 200 nM each of the primers R2_STRT_bulk and TRAC_I, TRBC_I, TRGC_I or TRDC_I and run with the same KAPA polymerase and cycling condition as the second PCR reactions. Each of the primers used in the third PCR contains a unique 6-nt barcode that identifies the sample origin, six random nucleotides at the start of R1 to ease cluster generation on Illumina sequencing chip, as well as portions of oligo sequences that are necessary for Illumina sequencing platforms. In the final library PCR reactions, 1.5 µL product from the third PCR reaction was used as template in total 20 µL reaction containing 200 nM each of the R1_library and R2_library primers containing sequences that completed Illumina sequencing adapters, 1x KAPA HiFi HotStart ReadyMix and run with the cycling condition of denaturation 2 min at 95 °C; 10 cycles of (15s of 98 °C, 30 s of 60 °C, 40 s of 72 °C); and final extension of 5 min at 72 °C.

The final library product from all samples were pooled separately for each TR chain and purified with 0.65x volume of AMPure XP beads (Beckman Coulter) according to manufacturer’s instructions. The purified products were eluted from the beads in 17 µL water, added with gel loading buffer and loaded directly onto a 1.2% agarose gel. Visible bands between 600–700 bp were excised out of the gel and purified using the Monarch DNA gel extraction kit (NEB). The gel-purified products were quality checked on BioAnalyzer (Agilent) and then submitted to 150 bp pair-end sequencing on ½ lane of an Illumina NovaSeq SP chip. Read 1, which has better overall quality, was used to sequence the CDR3 in the 3’−5’ direction starting from the first 26–46 bp of the 5’-end of the C genes. Read 2 sequenced the 5’-end of the V-genes starting from the 5’-UTR. The sequencing was performed at the Norwegian Sequencing Centre at the Oslo University Hospital.

### Analysis of T-cell receptor repertoires

The MiXCR^34^ pipeline was used for sequence mapping, assembly, and quantification, with the *analyze generic amplicon* preset and default parameters, but only functional chains were used in downstream analyses (parameter --only-productive). Additionally, since C genes cannot be identified due to sequence similarity, --dont-split-clones-by C parameter was used. Finally, since the whole receptor sequence could not be reconstructed due to short read lengths, --assemble-clonotypes-by CDR3 was used to collapse receptors based on their CDR3 nucleotide sequence. The reference database for MiXCR was formatted with RepSeqIO. Coordinates of CDRs and FR regions were manually incorporated due to the lack of similar reference sequences in the reference database. The immunarch^35^ package in RStudio^36^(R version 4.2.2^37^,) was used for the analysis of MiXCR outputs. Receptors with less than 5 assigned reads were discarded to reduce the effect of sequencing errors and improper assembly. Lastly, ggplot2 (3.4.4^38^,) facilitated visualization.

## Results

### Genomic organization of T-cell receptor loci

**Fig. 1:**
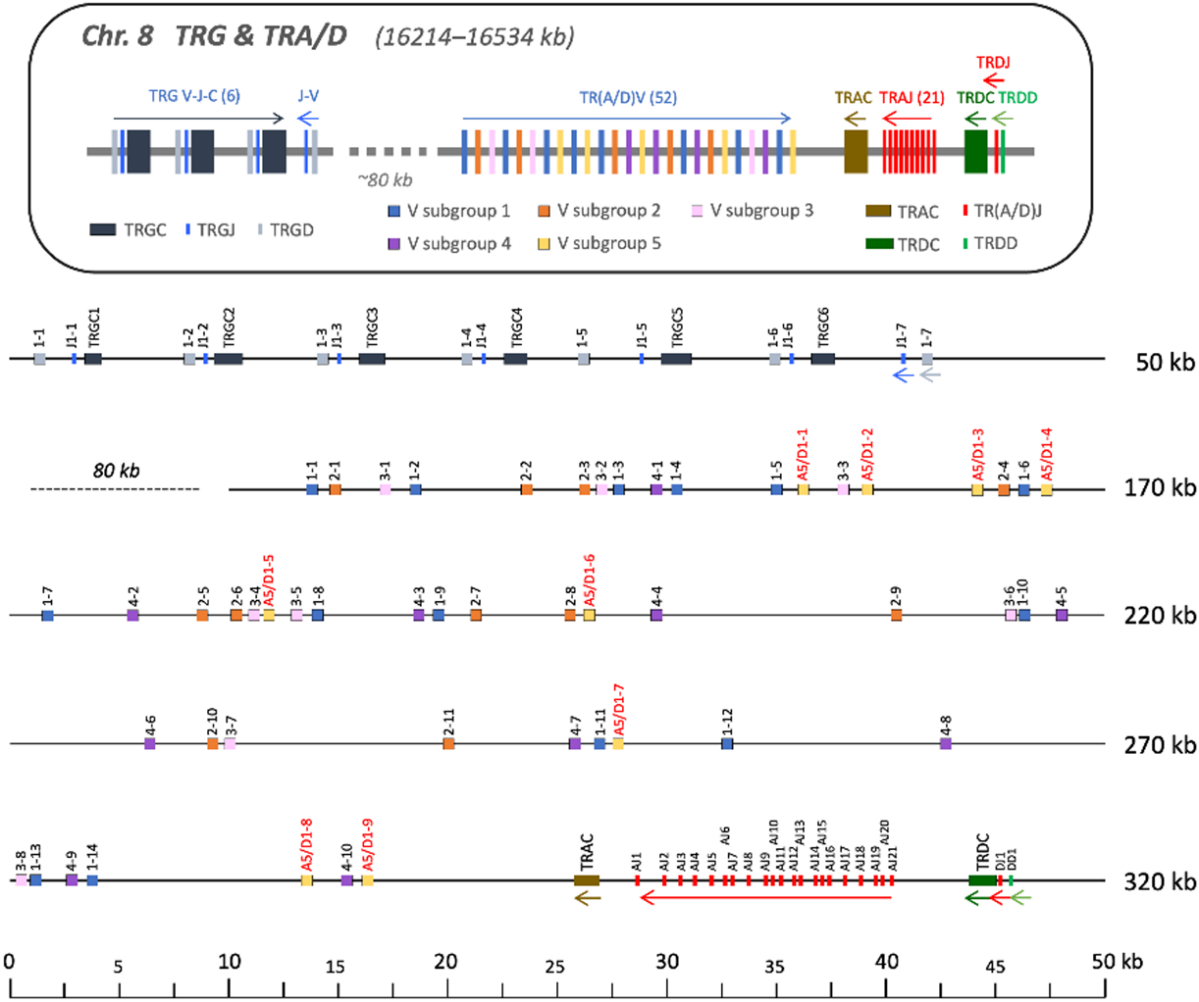
Schematic representation of TR gamma and TR alpha/delta locus in the Atlantic cod genome, chromosome 8. Top. Schematic map of the TRG and TRA/D loci on chromosome 8. The distances are not to scale. Numbers in the brackets after each gene indicate the total number of genes of that type. All V-genes are in the forward orientation whereas most J-, D-, and C-genes are in the reverse orientation as indicated by the arrows. Bottom. Genomic map of the TRG and TRA/D loci on chromosome 8 (16214–16534 kb). The V-genes are coloured according to the V-gene subgroup. D-genes are in red and J-genes are green. The numbers refer to V-genes unless otherwise specified. The first number indicates the V-gene subgroup and the last number is a running number for each individual V-gene subgroup. The V-gene subgroup 5 which is preferentially used by TR delta are denoted with «D» in the gene name and in red for clarity.

The T-cell receptor alpha and delta genes are co-located within a single translocon on chromosome 8, spanning a region between 16.3 Mb and 16.6 Mb (Supplementary Table 1, Fig. 1). This locus contains 52 V genes, all in the forward orientation, which could all participate in the recombination of TR alpha and delta chains. Immediately downstream of the V genes, a single alpha constant (C) gene is positioned in reverse orientation, followed by 21 joining (J) genes, also in reverse orientation. A single TR delta-C, J, and diversity (D) gene, all in reverse orientation, is located further downstream (Fig. 1). Gene functionality was assessed based on several criteria: the absence of stop codons or frameshifts, the presence of recombination signal sequences (RSSs) for V, D, and J genes, and the conservation of canonical sequence features. Based on these metrics, all but one of the V genes were deemed potentially functional. The exception, TRAV1-9*01, was identified as a pseudogene due to the absence of a complete CDR3 region and the canonical Cys104 at its 3’ end. However, it is noteworthy that both chains are non-productive in their germline configurations thus requiring insertion and/or deletion of nucleotide at the V-J or V-D-J junctions to generate productive chains.

Only 80 kb upstream of the TR alpha-delta locus, a distinct TR gamma locus is located. This locus comprises six complete and one partial mini-cluster within a 40 kb stretch (Supplementary Table 1, Fig. 1). Each complete mini-cluster includes a single V, J, and C gene in forward orientation, with an average size of 4 kb. The partial mini-cluster at the 3’ end, however, lacks a C gene and is oriented in reverse.

**Fig. 2:**
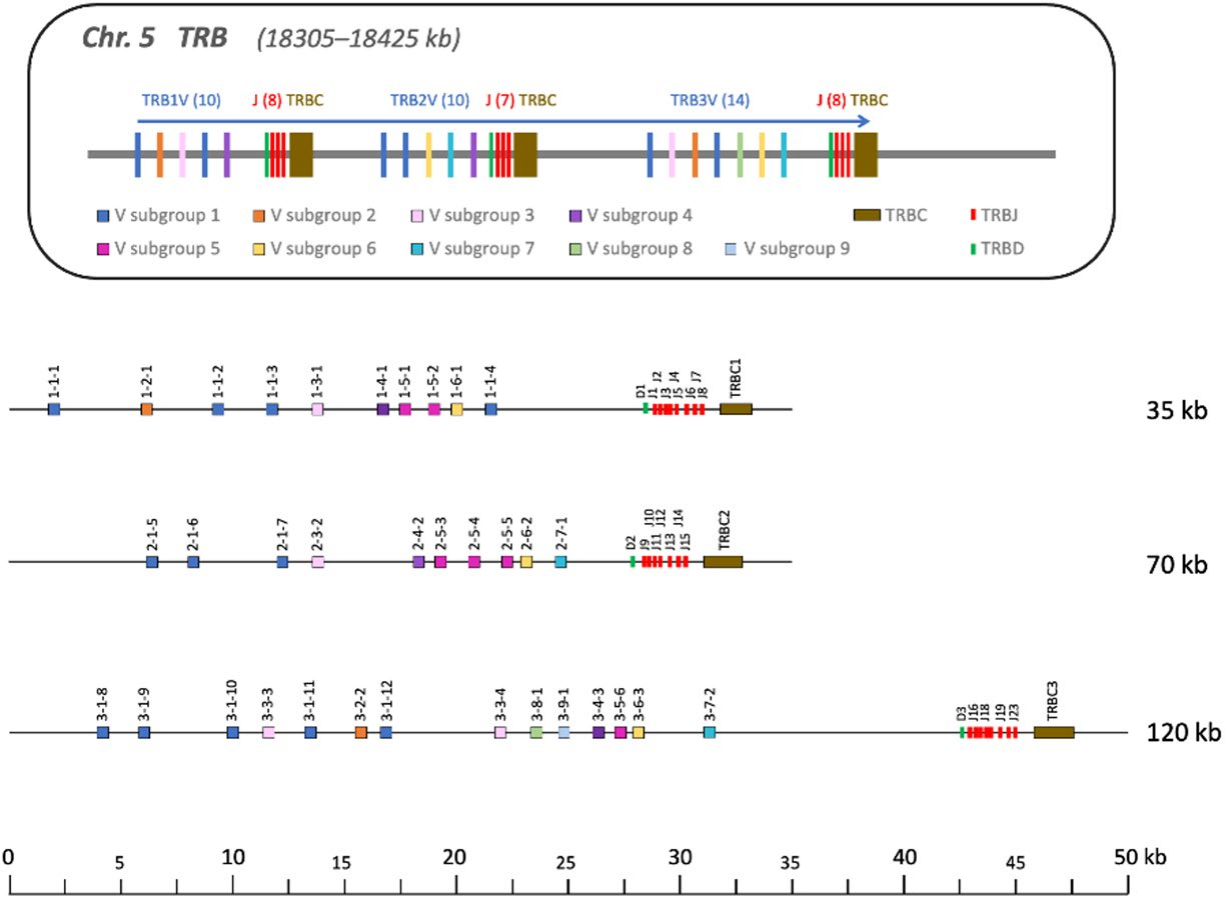
Schematic representation of TR beta locus in the Atlantic cod genome, chromosome 5. Top. Schematic map of the TRB locus on chromosome 5. The distances are not to scale. Numbers in the brackets after each gene indicate the total number of genes of that type. All genes are in the forward orientation indicated by the arrow. Bottom. Genomic map of the TRB locus on chromosome 5 (18305–18425 kb). The V-genes are coloured according to the V-gene subgroup. The number refers to V-genes unless otherwise specified. The first number inserted between TRB and V indicates the translocon, the second number indicates the V-gene subgroup, and the last number is a running number for each individual V-gene subgroup.

The TR beta chains are encoded on chromosome 5, beginning at 18.3 Mb, and are organized into three consecutive translocons, resembling the structure of immunoglobulin heavy chain locus^22^(Supplementary Table 1, Fig. 2). Each translocon contains a set of V genes, a single D gene, several J genes, and a single C gene, all in forward orientation. Translocons 1 and 2 are similar in size, approximately 30 kb each, while translocon 3 is about 10 kb larger. These translocons harbor 10, 10, and 14 V genes, respectively, all of which are functional. The J gene counts are also comparable across the translocons, with 8, 7, and 8 J genes identified in translocons 1, 2, and 3, respectively. Interestingly, the last J gene in each translocon exhibits distinct characteristics compared to the preceding J genes. These terminal J genes are notably longer, with an average length of 70 nucleotides, compared to the mean length of 50.4 nucleotides for the other J genes. Despite some differences in translocon size, TRB C genes are remarkably similar, where 144 of 175 amino acid residues are conserved between the three copies.

### TR genomic sequence features and classification

The nomenclature of T-cell receptor TR genes adheres to the IMGT format to ensure clarity and facilitate comparisons with other species, however, the dataset is not part of the IMGT database. Each gene was assigned a unique identifier, with chain types determined by initial similarity searches that showed clear homology to their respective counterparts in the IMGT reference dataset. However, gene identifiers were assigned based on genomic location, following a 5’ to 3’ orientation, rather than the historical order of discovery used for previously described genes, which lacked such genomic information. For TR beta V genes, an additional identifier was incorporated immediately after TRB to indicate their associated translocon, yielding identifiers such as TRB1V5-2 (translocon 1, V subgroup 5, gene 2).

**Fig. 3:**
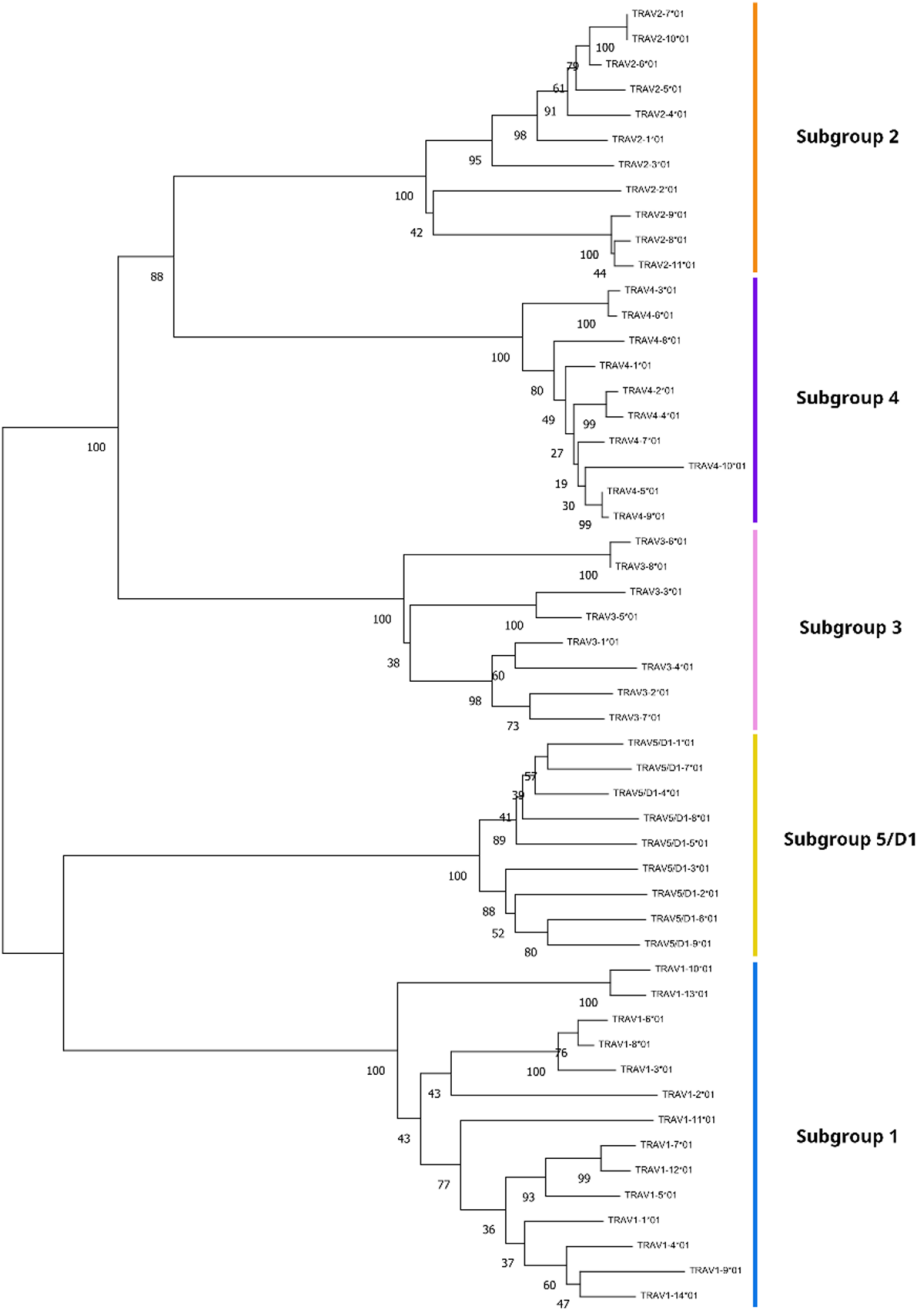
Phylogenetic tree of T cell receptor alpha/delta variable regions delineating V-gene subgroups. Numbers indicate the bootstrap values from 1000 replicates on the neighbour-joining tree. Evolutionary distances were computed using the Kimura 2-parameter method and are in the units of the number of base substitutions per site. The rate variation among sites was modelled with a gamma distribution (shape parameter = 5).

Phylogenetic analysis of translated coding regions of TR alpha/delta V genes identified five distinct gene subgroups, with sequence similarities over 80% within them (Fig. 3). Subgroup 1 was the largest, comprising 14 members, followed by subgroups 2 and 4, with 11 and 10 members, respectively. The TRA/D V genes displayed significant sequence variability, not only within the CDR regions but also in the framework regions (Supplementary Fig. 1A). RSS sequences exhibited strong conservation across subgroups in both the 7-mer and 9-mer motifs and spacer lengths. The canonical sequence CACAGTG was overwhelmingly represented in the 7-mer, and ACAAAAACC in the 9-mer. Spacer lengths were predominantly 22 bp across subgroups, except in subgroup 5. This subgroup displayed unique features, including a distinct 23-bp spacer with a divergent sequence and the 9-mer variant ACATAAACC, reflecting a possible functional or regulatory divergence.

For TR beta V genes, nine distinct gene subgroups were identified. These subgroups were generally less populous than TRA/D V families, with the largest, subgroup 1, comprising 11 members. Smaller subgroups, such as subgroup 6 and subgroup 7, contained only one member per translocon, and subgroup 8 and 9 were represented by a single member each in translocon 3 (Supplementary Fig. 2). Notably, the spatial arrangement of V genes from distinct gene subgroups showed strong conservation across the three translocons, suggesting relatively recent duplications of the entire translocon. Similarly to the TRA V genes, TRB V genes exhibited substantial sequence diversity that extended beyond the CDR regions (Supplementary Fig. 1B). RSS sequences were conserved within subgroups but demonstrated subtle yet consistent divergence between subgroups in the 7-mer, 9-mer, and spacer regions. Spacer lengths varied, with half of the subgroups having a 22-bp spacer and the other half a 23-bp spacer. These differences suggest a tightly regulated recombination, tailored to specific V gene subgroups.

In contrast, the TRG V genes showed significantly less diversity. Using an 80% sequence similarity threshold, all TRG V genes fell into a single subgroup (Supplementary Fig. 3). Sequence diversity was moderate and was concentrated in the CDR regions (Supplementary Fig. 1C). Their RSS sequences were also highly conserved, apart from TRGV1-5, which displayed a markedly different and non-canonical RSS. This unique feature may indicate limited recombination potential or a specialized role for this gene within the repertoire.

### Characterization of TR repertoire by deep sequencing

**Fig. 4:**
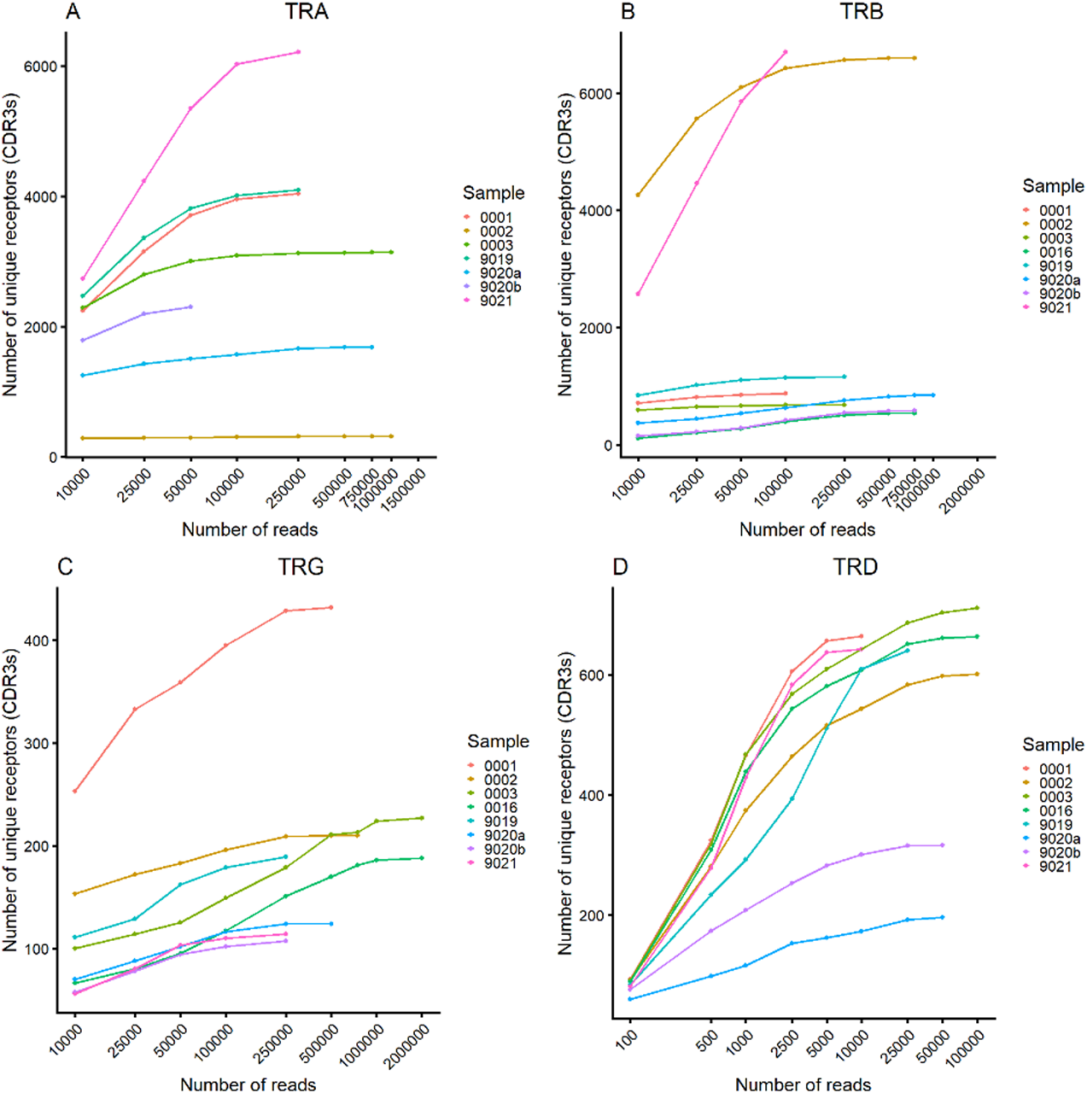
Number of unique receptors based on sequencing depth and abundance-based receptor rank and diversity for T cell receptor chains. Number of unique receptors identified at the CDR3 amino-acid sequence level in relation to mapped sequencing reads for TR alpha **(A)**, TR beta **(B)**, TR gamma **(C)** and TR delta **(D)** receptors. Subsamples of reads were created from mapped reads for each sample. Sampling depth was adjusted for each sample according to total number of mapped reads.

To investigate the TR repertoire of Atlantic cod, spleen samples were collected from seven fish (six for TR alpha). For each TR chain, two technical replicates were included for one of the fish (identifiers F9020a and F9020b). cDNA was PCR-amplified using a 5’RACE protocol with primers specific to the constant (C) gene, and the primers in the final PCR were positioned immediately downstream of the J-C junction. Each TR chain was targeted with unique, specific primers, and sequencing was performed on the Illumina HiSeq platform. Details on primers and sequencing statistics are provided in Supplementary Tables 2 and 3. We analysed the effects of sequencing depth on the number of unique receptors to gauge how well the repertoire diversity is captured in our spleen samples. Most samples for TRA, TRB and TRD show clear signs of saturation of the sampled repertoire at the sequencing depths used, with 1 million reads being sufficient for all chains. TRG, due to its smaller diversity, was sufficiently covered by 100,000 reads (Fig. 4).

TR chains were assembled at the CDR3 level, and the number of unique functional sequences was assessed. On a nucleotide level, the diversity of unique functional sequences was comparable across most TR chains. TR alpha exhibited the highest diversity, with a median of 4,422 unique sequences, followed by TR beta, with a median of 852. Surprisingly, TR delta showed a comparable level of diversity, with a median of 642 unique functional sequences. In contrast, the TR gamma chain was markedly less diverse, with a median 196 unique CDR3s. Since we only sampled part of the spleen, these numbers provide a lower bound estimate of the total diversity present in the whole organ. And while unlikely, it is theoretically possible to have minimal overlap between the TR sequences in the rest of the organ, hence the upper bound median diversity is estimated to be 29,480 for TRA, 5,680 for TRB, 4,279 for TRD and 1,306 for TRG chains respectively.

Leveraging the technical replicates, we can also further investigate the total repertoire size. When comparing samples F9020a and F9020b, we found that of the 3,053 unique TRA CDR3s identified in the two samples, 1,542 were shared among replicates. Similar ratios were found for TRB (464 of 975) and TRG (79 of 165), suggesting that the real repertoire size may be closer to the lower bound estimate of the repertoire size. TRD on the other hand showed very low overlap, only 6 of 508 CDR3s were shared between technical replicates, indicating that the real repertoire size is most likely close to the upper bound of the estimated repertoire size. In order to further explore similarity between replicates, we calculated rank-correlation of overlapping sequences for all replicates of all chain types. Highest correlation was found for TRA (Kendall’s τ = 0.66, *P* < 10^− 15^), followed by TRG (τ = 0.28, *P* = 0.0003). TRB and TRD showed no significant correlation (τ = 0.05, *P* = 0.11 for TRB and τ=−0.39, *P* = 0.22 for TRD respectively).

While the total number of unique functional sequences varied only moderately between chain types except gamma, the fraction of functional sequences revealed a striking difference in the case of TR delta. For all other chains, the fraction of functional sequences was on average above 75%. However, for TR delta, this value was significantly lower, averaging only 11%. Despite a comparable final count of functional TR delta sequences to TR alpha and beta, the total number of TR delta sequences produced to achieve this count was substantially higher.

**Fig. 5:**
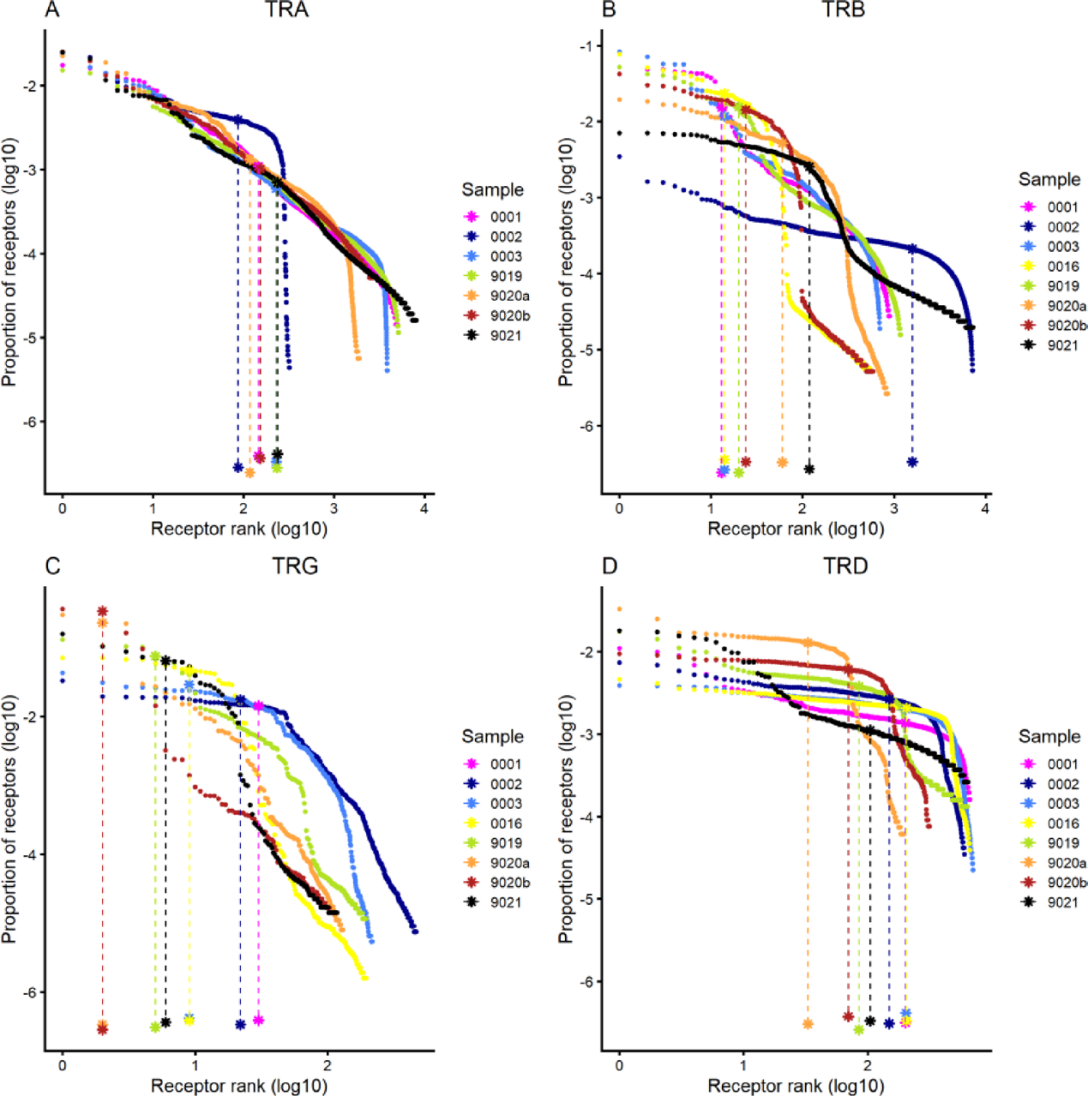
Abundance-based receptor rank and proportion of repertoire of TR alpha **(A)**, TR beta **(B)**, TR gamma **(C)** and TR delta **(D)** receptors. Each point represents an individual receptor, stars denote D50 values for each sample. All axes are logarithmic for better readability. D50 values and corresponding horizontal lines were jittered slightly for better visibility.

To further characterize the TR repertoires, we calculated D50 values, which indicate the proportion of top unique receptors required to cover 50% of the total expressed repertoire. For all TR chain types, 2.7–5.4% of the topmost used receptors accounted for 50% of the repertoire, irrespective of the total diversity, suggesting a similar repertoire structure (Supplementary Table 4) biased towards a few sequences. However, absolute diversity differed among chain types. The median D50 values for TRA, TRB, and TRD were 172, 231, and 120, respectively, whereas TRG chains exhibited much lower diversity with a median D50 value of 11. Notably, sample F0002 displayed an unusually diverse and even TRB repertoire, requiring nearly 20% of the top receptors to reach 50% coverage (Fig. 5). This was reflected in the Shannon diversity index, where F0002 exhibited a TRB diversity of 12.2, substantially higher than the average of 7.9 for this chain. Overall, TRA chains had the highest average Shannon diversity at 9.7, followed by TRD at 8.3. TRG chains showed significantly lower diversity, with an average Shannon index of 4.6 (Supplementary Table 4).

CDR3 length analysis revealed similarities between TRA and TRB chains, with averages of 12.5 and 12.3 amino acids, respectively. This similar length may result from the longer J genes in TRA compared to TRB, as the last, significantly longer TRBJ gene in each translocon is unused. TRG chains displayed slightly shorter CDR3s, averaging 11 amino acids, while TRD chains had longer CDR3s, averaging 13.9 amino acids. The CDR3 length distribution is also wider for TRD than for other chains with a larger fraction of short and long CDR3s (Supplementary Fig. 4).

**Fig. 6:**
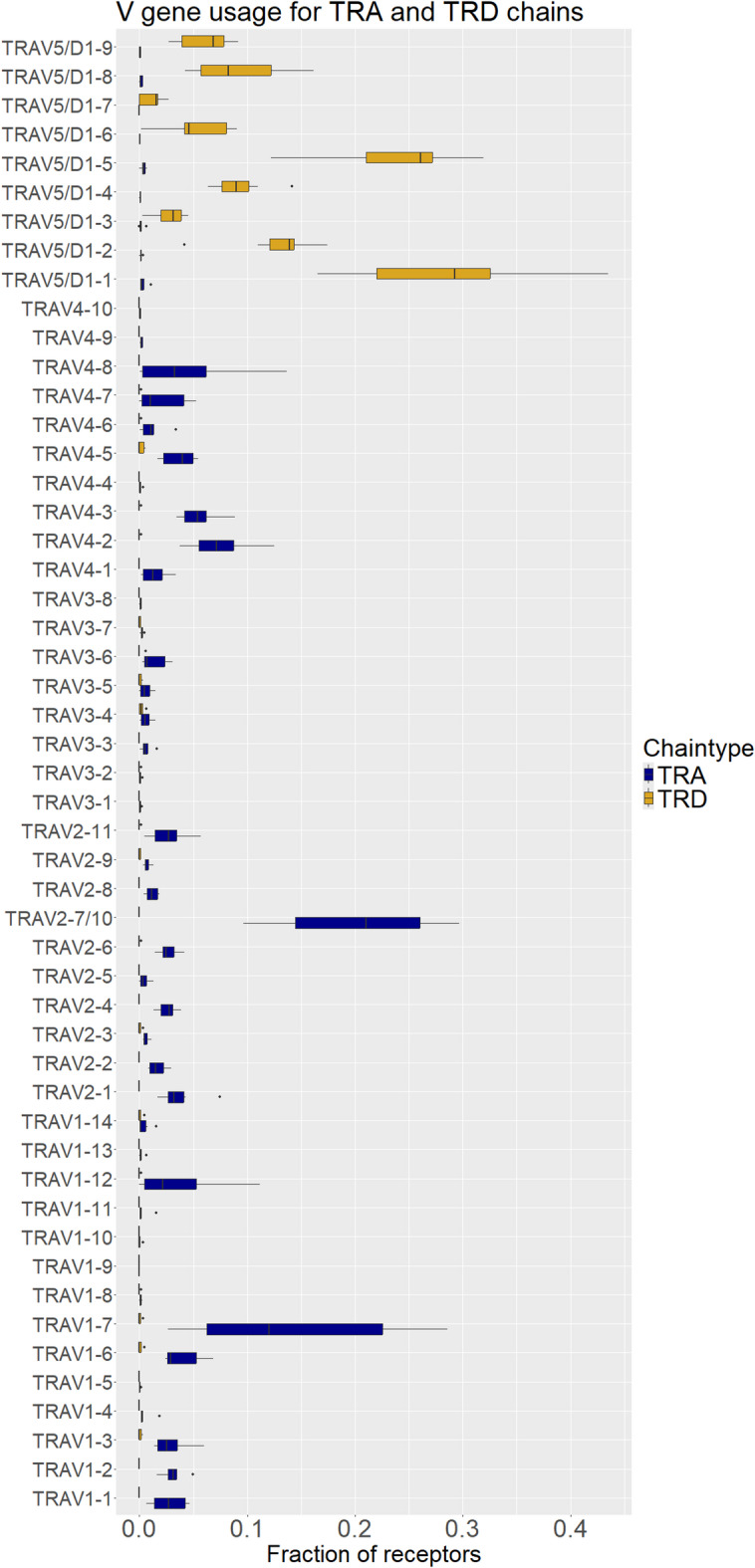
V gene usage for TR alpha (blue bars) and delta (golden bars) chains. Genes are ordered based on their subgroup name and individual gene name. One sample was used from each fish in the calculation (no technical replicate) of gene usage, error bars represent standard deviation between the different fish.

Next, we performed V-gene usage analysis for each chain. Due to the limited sequencing length and the high similarity of V gene sequences, in some cases V genes could not be unambiguously assigned. The frequency of this event was 24.1% and 4.4% for TRA/TRD chains respectively, 1.2% for TRB and 1.3% for TRG. The high ambiguity found in TRA V genes can be attributed to pairs of highly similar, but not identical V gene pairs, such as TRAV2-7 and TRAV2-10, TRAV3-3 and TRAV3-5, TRAV4-3 and TRAV4-6, TRAV1-12 and TRAV1-7, TRAV4-5 and TRAV4-9. The ambiguously mapped sequences were removed from the analysis; hence the usage of the nearly identical V gene pairs is expected to be higher than measured. V-gene usage analysis revealed selective gene usage for TR alpha and delta chains. Subgroups 1 to 4 were predominantly used for TR alpha chains, while subgroup 5 was almost exclusively used in TR delta chains, leading to its designation as TRAV5/D1 to reflect this specificity (Fig. 6). While all four subgroups contributed to TR alpha sequences, 13 of the 34 TRA V genes showed no substantial expression in any sample. TRA V-gene usage was robust across fish, with an average pair-wise correlation coefficient of 0.69. In contrast, TRD V-gene usage was more balanced, with all 9 V genes expressed and a higher correlation coefficient of 0.9. For TRB chains, all V genes were expressed; however, inter-fish differences were more pronounced, with an average correlation coefficient of only 0.53 (Supplementary Fig. 5). To investigate inter-translocon recombination in TRB, we first identified the minor fraction of sequences in which the J genes could be unambiguously identified, which due to the high sequence similarity of the J genes, constituted only 18% of all TRB sequences. Despite this, we identified 1,193 sequences across all samples that showed clear evidence of inter-translocon recombination, 6.2% of the total TRB sequences. Notably, long TRBJ genes (TRBJ8, TRBJ15 and TRBJ23) were not used in any sample. TRG V-gene usage showed a preference for TRGV1-2 and TRGV1-4, while TRGV1-3 and TRGV1-7 were used infrequently, the latter potentially due to its inversed orientation in the genome. Despite the non-canonical RSS sequence of TRGV1-5, it is still used in 15% of the repertoire on average, highlighting flexible signal recognition during recombination. Expression patterns were consistent across samples, with an average correlation coefficient of 0.86 (Supplementary Fig. 6).

**Fig. 7:**
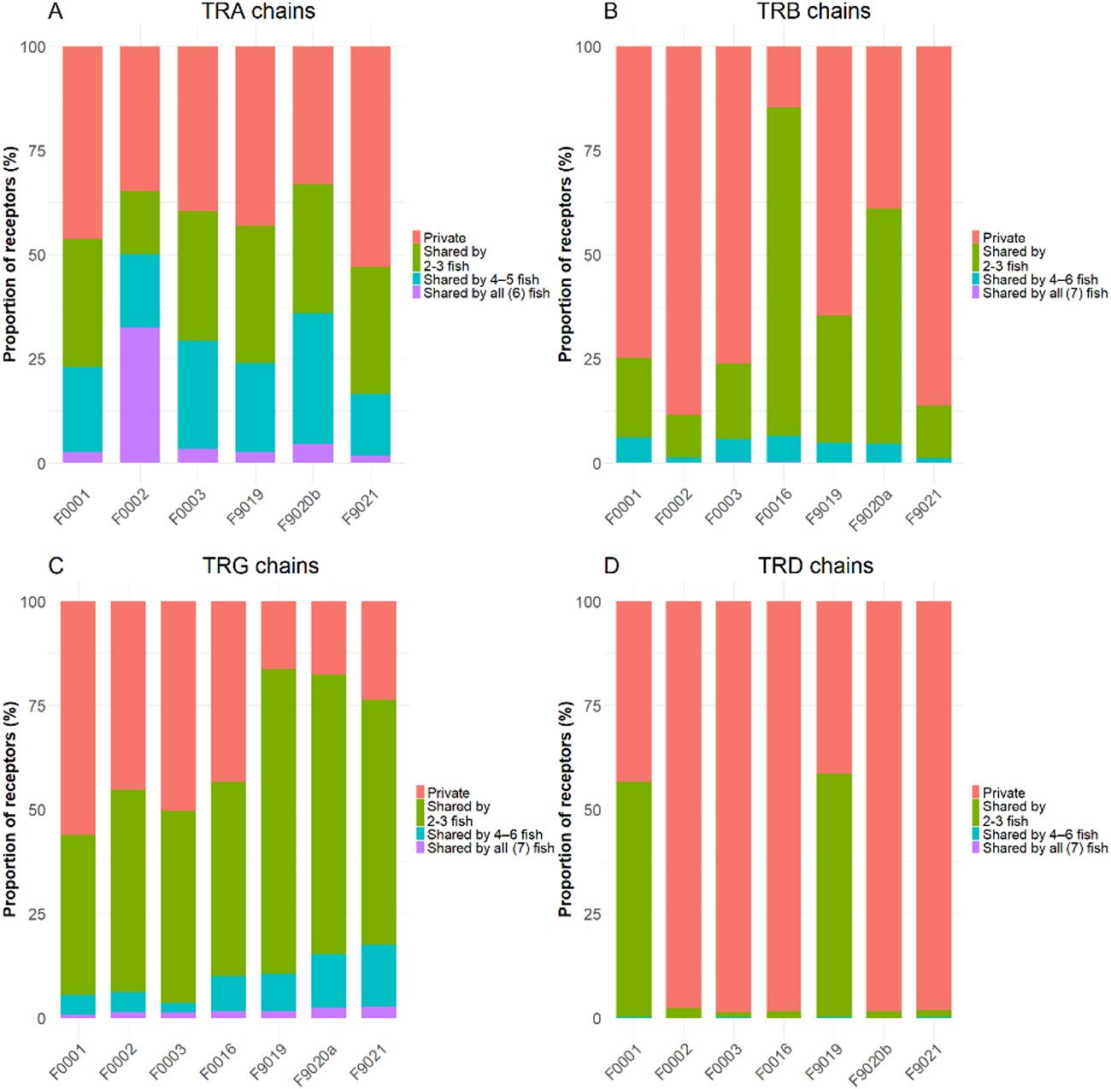
Proportion of the repertoire unique to each fish (private) and the proportion of the repertoire shared between two and widely shared between several fish per T cell receptor chain. Colours denote the proportion of the levels of sharedness of the repertoire.

Finally, we assessed receptor sharedness, also known as public receptors, on an amino acid level among samples for each chain (Fig. 7). In TRA and TRG repertoires, 7.8% and 1.2% of CDR3 sequences, respectively, were shared across all samples. Fish F0002 exhibited a particularly high use of public sequences (32%) for TRA chains, likely due to its low total unique receptor count. Conversely, 41.7% of the TRA repertoire and 38.2% of the TRG repertoire were private, despite their differing diversity. For TRD chains, no sequence was shared across all fish. Additionally, no functional sequence was shared by more than four fish. TRD samples fell into two categories: those with large private repertoires (over 90%), except for F0001 and F9019 with lower private fractions, resulting in an overall average of 74.7%. On average 6.33% of TRB CDR3s were shared among samples, while 59.7% were private.

## Discussion

The Atlantic cod represents a compelling model in vertebrate immunology, characterized by the loss of MHC class II, CD4^20^, and a non-functional AID^39^, which fundamentally reshapes its adaptive immune landscape. The complete absence of CD4⁺ T cells is expected to drive a shift from humoral to cellular immunity, as evidenced by the species’ weak T cell dependent antibody responses. A robust T-cell response is contingent on a diverse expressed repertoire^40^, suggesting at least a moderate effective diversity in the Atlantic cod. On the other hand, the higher number of expressed MHC I molecules has been shown to decrease T-cell diversity in the rodent bank vole through T cell depletion during negative selection^41^. The Atlantic cod has a highly expanded set of MHC I genes, although some showing non-classical MHC II-like motifs for cross-presentation^42^, suggesting a potential negative selection on the size and diversity of the expressed T-cell receptor repertoire.

In this study, we have completed the full genomic annotation of all TR loci in Atlantic cod, making it one of the few fish species with a comprehensive TR genomic framework. TR receptor sequencing elucidated the identity of TR delta V-genes. Additionally, we have established the baseline TR repertoire for all four chains in untreated fish, providing a crucial reference for future studies investigating immune responses to infection and vaccination. While previous research offered partial insights, a comprehensive analysis integrating both genomic and expressed repertoire data was lacking. The groundwork established here not only advances our understanding of Atlantic cod T-cell responses but also lays a critical foundation for future high-throughput TR repertoire analyses, enabling deeper exploration of alternative adaptive immune strategies in this unique species.

The TR alpha/delta locus of the Atlantic cod follows the conserved locus organization generally observed in fish. With 52 V and 21 J genes in total, the locus is among the smaller ones observed so far in fish. There are only 5 V gene subgroups and nearly all genes are functional. Even though V genes show some sequence variability, this puts the Atlantic cod on the lower end of the scale in regards to germline gene diversity among described species, far below the Atlantic salmon^7^, rainbow trout^8^, channel catfish or zebrafish^9^. This is consistent with expressing a smaller T-cell receptor repertoire in response to the substantial expansion of MHC class I. On the other hand, the lack of deletions and frameshifts suggest that the Atlantic cod maintains a smaller but functionally important set of alpha and delta chains. This is also supported by the highly conserved V gene subgroup-specific RSS signals and selective gene usage in the individual repertoires. An interesting facet of alpha/delta locus organization is the possibility of simultaneous expression of both chains due to the recombination of inverted gene segments retaining the whole locus for secondary rearrangements. This is in contrast to human T cell development, where TR delta genes are excised as alpha-beta T cells mature^43^. This process can theoretically result in secondary rearrangements, producing both TR alpha and delta chains on the same allele. Notably, an analysis of previously published single-cell transcriptomic data of the Atlantic cod revealed that 121 out of 1958 alpha-beta T cells (6%) expressed both TR alpha and delta C genes (LOC115548821 and LOC115548656, respectively)^33^. However, the functionality of these chains could not be established with the single-cell sequencing protocol used in that study.

The genomic organization of TR beta loci generally follows a translocon structure common in fish, with multiple V genes preceding clusters of D and J segments, usually ending in a single C gene. However, the germline diversity of TRB chains is highly variable across species, influenced by evolutionary events such as whole-translocon duplications, as observed in zebrafish^12^ and large yellow croaker^13^, or whole-genome duplications, as seen in salmonids^14^. The Atlantic cod also has its own peculiarity in the architecture of the TR beta locus with three consecutive triplicates of the translocon. Moreover, the three translocons show high similarity, with V gene subgroups organized in similar order within translocons, suggesting recent duplications. Of the 9 V gene subgroups, two were unique to the 10 kb longer translocon 3, the rest were shared. Consequently, the 34 V and 23 J genes, although recently expanded, still represent a moderately sized germline repertoire for TR beta chains, with half as many V genes as salmon^14^ or the channel catfish^10^. The Atlantic cod shows high sequence similarity in its C genes, most likely due to the recent translocon duplication, however, the three copies may provide ample resources for future subfunctionalization or become pseudogenized.

The TR gamma locus exhibits fundamental differences in genomic organization, with species displaying either a mini-cluster arrangement or a translocon structure. In the mini-cluster organization, observed in “mouse type” TRG loci, genes are arranged in multiple small, tandemly repeated units, each containing one or more V genes, a single J gene, and a C gene^16^. In contrast, the “human type” organization follows a more conventional translocon structure^17^, with V genes preceding a contiguous set of J and C genes. Despite this structural flexibility, TRG genes exhibit the lowest overall genomic diversity among TR loci. The TR gamma locus of the Atlantic cod is also by far the smallest among the different chain types, consisting of 6 mini-clusters with a single V, J and C and a partial cluster missing a C gene, in reverse orientation. This is a comparable germline diversity to other described TR gamma loci in fish.

While large-scale TR repertoire analyses in fish remain scarce, establishing a baseline for diversity and gene usage allows us to assess whether the Atlantic cod exhibits an expanded repertoire or displays unusual expression patterns. Comparative data from other fish species suggest considerable variability in TR repertoire organization. In zebrafish, TR beta diversity is limited, with a predominantly public repertoire^44^, while TR gamma-delta diversity is extremely constrained. In contrast, Atlantic salmon expresses a more diverse TR repertoire, with 652 unique sequences identified from 759 cDNA clones, with more TR alpha and delta sequences than zebrafish^7^. In rainbow trout, spectratyping analyses revealed a balanced and largely private repertoire in naïve fish, with clonal expansion occurring only upon viral infection^45^. Our findings reveal a diverse repertoire in Atlantic cod, with comparable number of unique TR alpha, beta, and delta receptors, despite TR delta being constrained to only nine delta-specific V genes. The deep and targeted sequencing of repertoires revealed thousands of unique, functional receptors for TR alpha, beta and delta chains and hundreds of TR gamma from spleen samples containing less than 10^7^ T cells. The resulting total receptor diversity can conservatively be estimated to be in the millions of unique functional alpha-beta receptors and hundreds of thousands of gamma-delta receptors in the Atlantic cod. Repertoire diversity could be up to two orders of magnitude higher if there is spatial heterogeneity in the spleen T cell populations, which in the case of gamma-delta receptors would be much higher than previously observed. The more diverse TRD repertoire, in conjunction with its longer CDR3 sequence also suggests that TRD is the more prominent chain in antigen recognition compared to TRG. CDR3 lengths fell within ranges reported for other fish. TRA and TRB averaged 12.5 and 12.3 amino acids, slightly longer than Japanese flounder^46^(11.2 for both chains) and zebrafish TRB(12.0) but not zebrafish TRA^47^(13.1), and clearly longer than rainbow trout TRB^45^(8) or human and mouse^48^. TRG and TRD CDR3 lengths were also comparable to other fish: TRG was shorter (mean of 11 amino acids), while TRD averaged 13.9 amino acids, marginally longer than Japanese flounder^46^. TRG CDR3s were longer than those in humans and mice (means 7.2 and 8.8), whereas TRD was only longer than mouse TRD (means 14.5 and 12.7 for human and mouse, respectively). Overall, CDR3 sequences showed wider length distributions in the Atlantic cod than other fish described before, however, sequences at the ends of the length distribution were very rare, hence they may just be missing from previous studies with less sequencing depth.

V-gene usage of TRAV genes shows that only around half of them were expressed in unchallenged fish, suggesting a substantial untapped pool of additional diversity. Notably, both TR alpha and delta chains require junctional insertions or deletions to form functional proteins, which is a mechanism that inherently increases repertoire diversity through the random nature of these processes, while still using a limited number of germline genes. The existence of a larger and diverse repertoire for alpha chains is not entirely surprising; however, the diverse TR delta repertoire suggests a strong capacity for antigen recognition, through which a robust mucosal response may be developed. Additionally, a very high fraction of non-functional TR delta chains was detected, which can have several explanations that are not mutually exclusive. First, some of the delta chains may be expressed in alpha/beta T cells. It is assumed that TR delta chains recombine prior to alpha-chain recombination during thymocyte development. In this case, a thymocyte with a non-functional delta recombination event may subsequently recombine the alpha and beta loci. Alternatively, it has been shown that some gamma-delta T cell populations can survive and mature without positive selection^3^ and some of these cells might reach the spleen with a non-functional receptor. Confirming this scenario would require a more comprehensive, single-cell investigation across multiple tissues. Finally, previously published single-cell transcriptomics data^33^ showed a substantial portion, approximately 40%, of cells in the gamma-delta T cell cluster expressed TR beta chain in addition to TR gamma and delta. Thus, it is an intriguing possibility that non-functional delta chains can be rescued and replaced by the simultaneous expression of a functional TR beta, resulting in the potential pairing and surface expression of an unconventional gamma-beta TR. The expression of a similar, non-canonical receptor consisting of beta and delta chains have been confirmed on the protein level in the DND41.C1 human cell line^49^, suggesting that there may exist a small population of T cells with unusual pairing of TR chains. It is also noteworthy that there is almost no overlap between V gene usage of TRA and TRD chains, despite no obvious difference outside the conserved, family specific RSS signals of subgroups 1 to 4 and subgroup 5. This indicates that the Atlantic cod RAG proteins can distinguish between TRAV and TRDV genes during the maturation process of T cells. Despite technical limitation, we found clear evidence of recombination across translocons, of a TRB V gene from one translocon recombining with J gene from a different one, showing that the unusual translocon organisation does not limit full utilization of possible V-J pairing. It also suggests that no subfunctionalization happened after the translocon duplication, unlike what is suggested from the alternative splicing pattern found for the two TRB translocons in zebrafish^12^.

Finally, our data indicate that shared repertoires are largely confined to TR alpha and TR gamma chains, whereas TR beta and delta receptors remain overwhelmingly private. The reason for the existence of such an idiosyncratic T-cell receptor repertoire is unclear; however, correlation of V-gene usage was also the lowest for TR beta among different fish. In the case of TR alpha-beta, canonical model of TR binding to peptide-MHC assigns TR alpha as primary contact with MHC I scaffold whereas TR beta makes most contribution in specific binding to the antigenic peptide^50^. The shared TR alpha repertoire and diverse and private TR beta repertoire in cod is compatible with this model. As for TR gamma-delta, the significantly longer CDR3 of the delta chain (13.9 vs. 11 amino acids for TR gamma) suggests possibly more contribution of TR delta in antigen binding, thus its relatively high diversity. This seems to suggest an alternative approach to repertoire diversification to TR delta chains, which also have very few public chains, but highly correlated V-gene usage between individuals. For TR gamma, this is likely attributable to its limited germline diversity, aligning more closely with rainbow trout, which exhibits predominantly private TR repertoires, rather than Atlantic salmon, where larger fraction of the repertoires appears to be public. These findings underscore that the tug-of-war between different evolutionary forces shaping the T cell receptor repertoire yielded a surprisingly diverse and individual-specific repertoire, in line with the need for a robust T cell response.

## Supplementary Information

Below is the link to the electronic supplementary material.

Supplementary Material 1

Supplementary Material 2

Supplementary Material 3

Supplementary Material 4

Supplementary Material 5

## Data Availability

Sequencing data will be available from EBI in the ArrayExpress collection in BioStudies, submission number E-MTAB-15001. [https://www.ebi.ac.uk/biostudies/ArrayExpress/studies/E-MTAB-15001?key=bea62d5f-9fc1-42a8-a401-597a2846b637].
