# Supplementary material for: Characterization of T-cell receptor loci and expressed repertoire reveals a capacity for robust T-cell response in Atlantic cod (Gadus morhua)
Source: Sci Rep. 2026 Mar 22;16:14483. doi: 10.1038/s41598-026-45018-x (PMC13149839; doi:10.1038/s41598-026-45018-x)
Supplement: Supplementary file 5 — Supplementary Material 5 [file 41598_2026_45018_MOESM5_ESM.pdf]

Supplementary Material

Supplementary figure 1

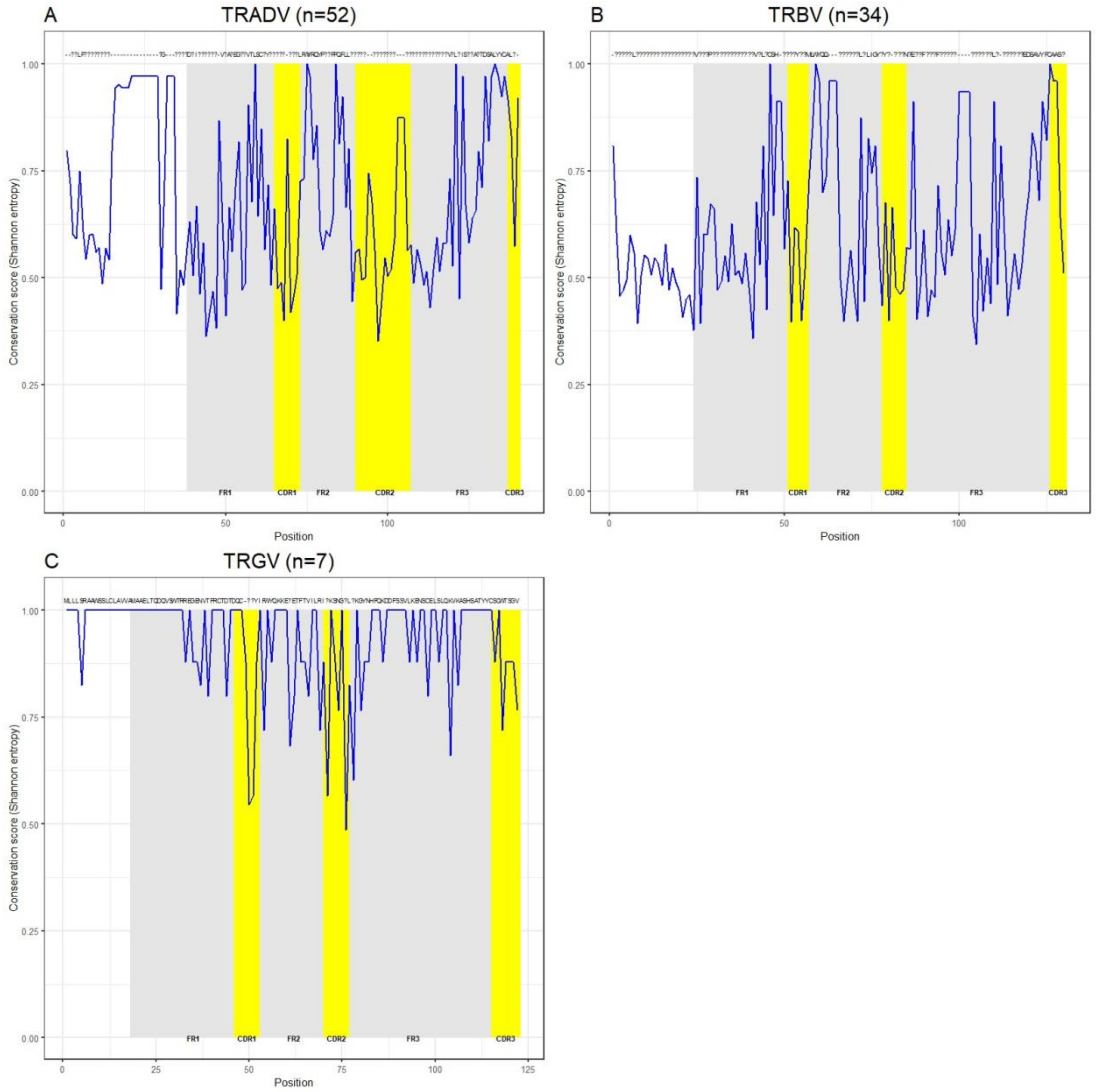

Shannon entropy indices for T cell receptor germline gene segments. Yellow and grey fields represent CDR and framework regions, respectively. Letters on top denote the consensus sequence, dashes (-) are gaps, question marks (?) are too diverse to set consensus. Number in parentheses on top of each figure is the number of V genes in each group. A) T cell receptor alpha/delta V genes, B) T cell receptor gamma and C) T cell receptor beta V genes.

Supplementary figure 2

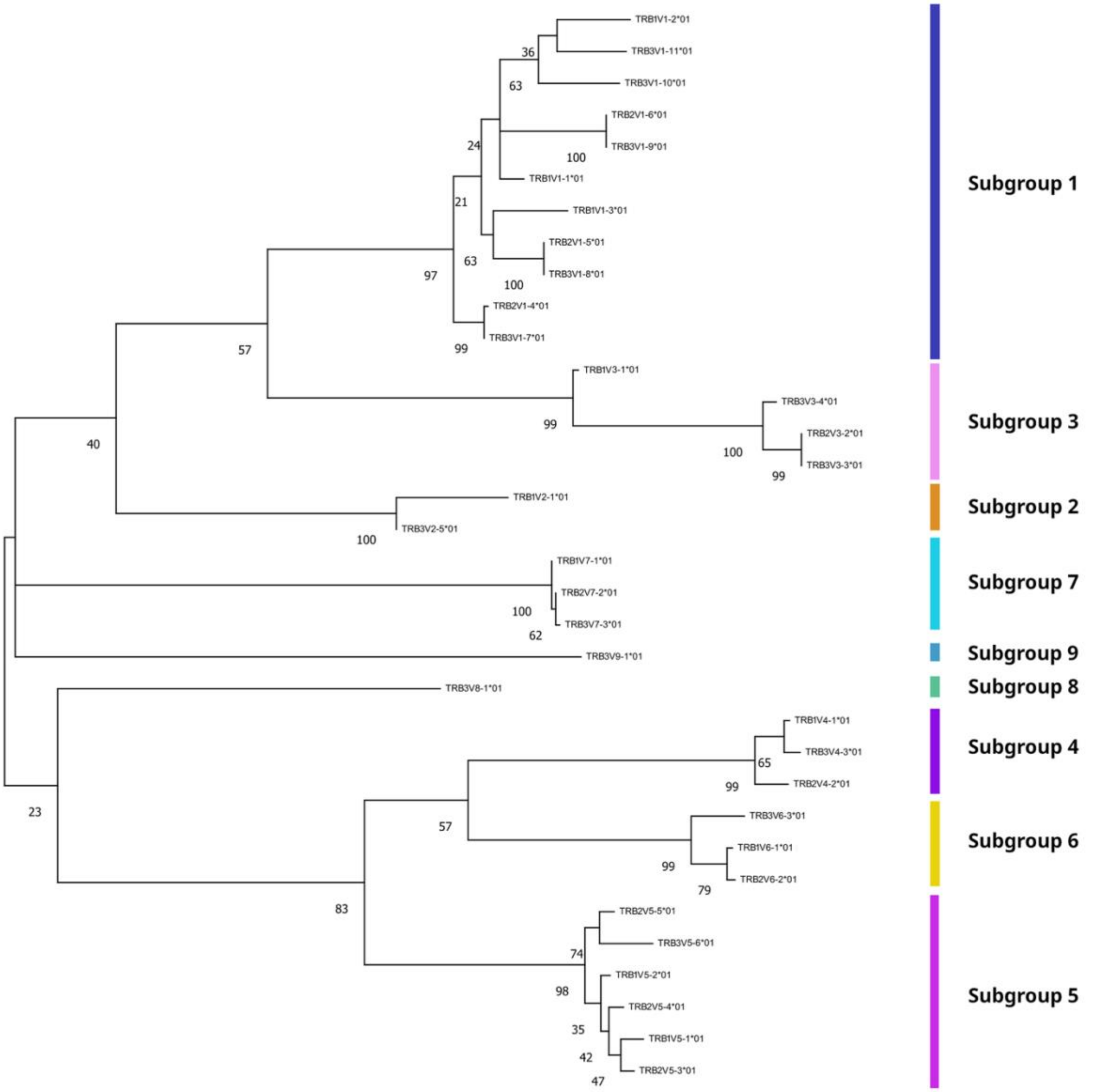

Phylogenetic tree of T cell receptor beta variable regions delineating V-gene subgroups. Numbers indicate the bootstrap values from 1000 replicates on the neighbour-joining tree. Evolutionary distances were computed using the Kimura 2-parameter method and are in the units of the number of base substitutions per site. The rate variation among sites was modelled with a gamma distribution (shape parameter = 5)

Supplementary figure 3

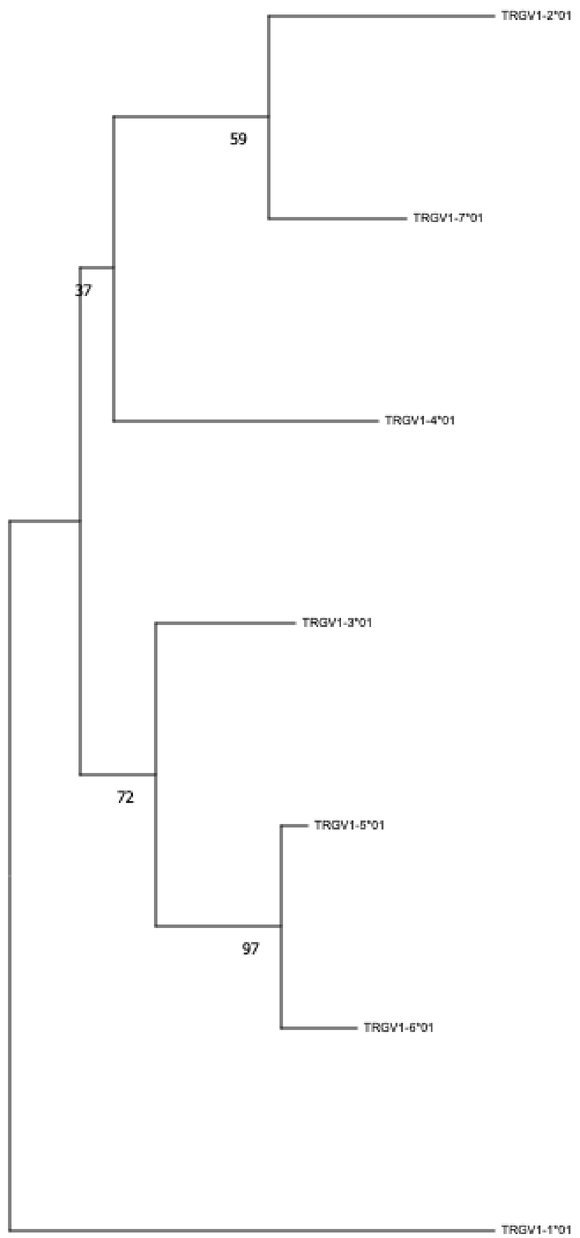

Phylogenetic tree of T cell receptor gamma variable regions delineating V-gene subgroups. Numbers indicate the bootstrap values from 1000 replicates on the neighbour-joining tree. Evolutionary distances were computed using the Kimura 2-parameter method and are in the units of the number of base substitutions per site. The rate variation among sites was modelled with a gamma distribution (shape parameter = 5)

Supplementary figure 4

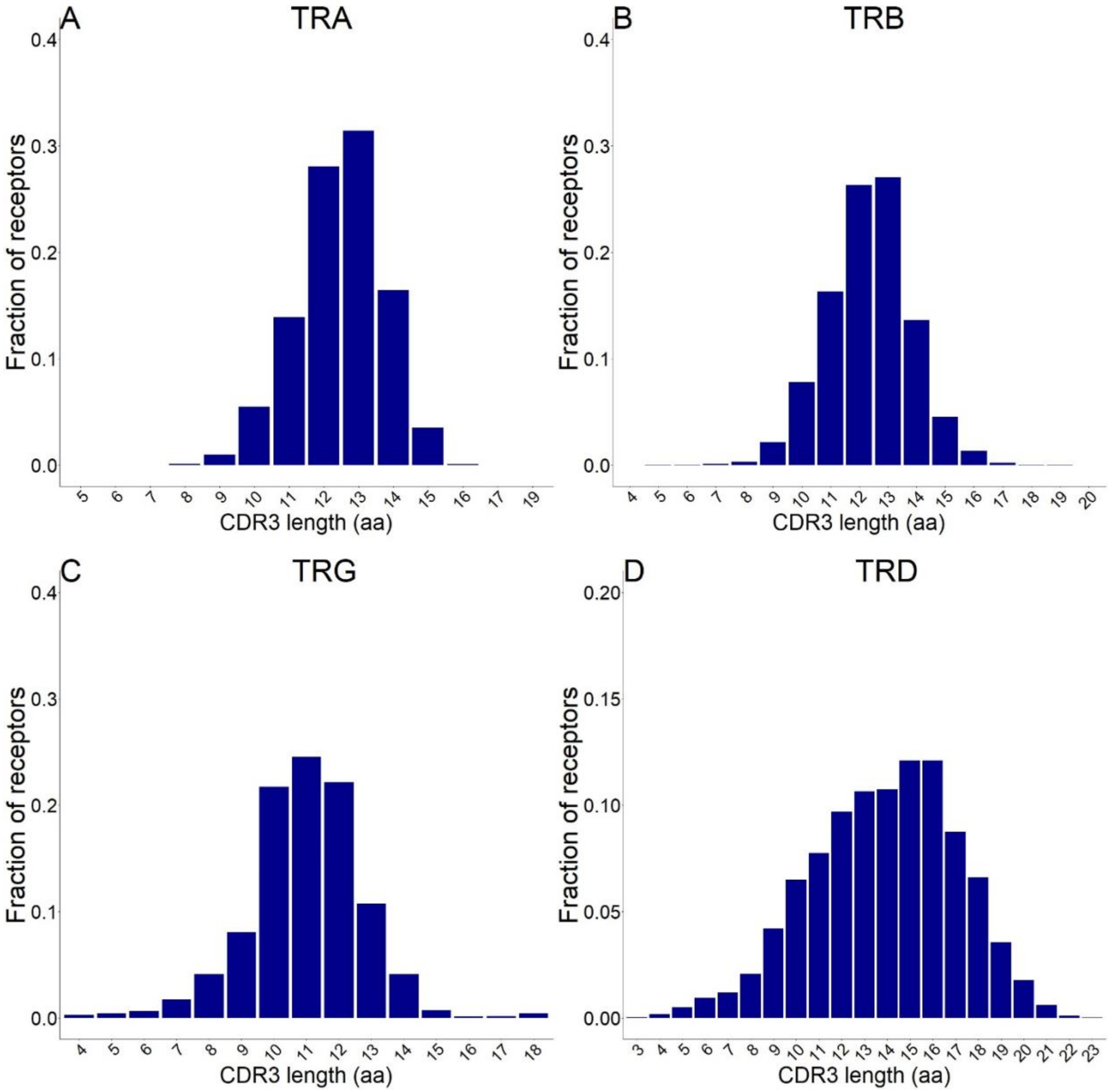

CDR3 length distribution of T cell receptor alpha (A), beta (B), gamma (C) and delta (D) chains. All samples were combined for calculating CDR3 length distributions.

Supplementary figure 5.

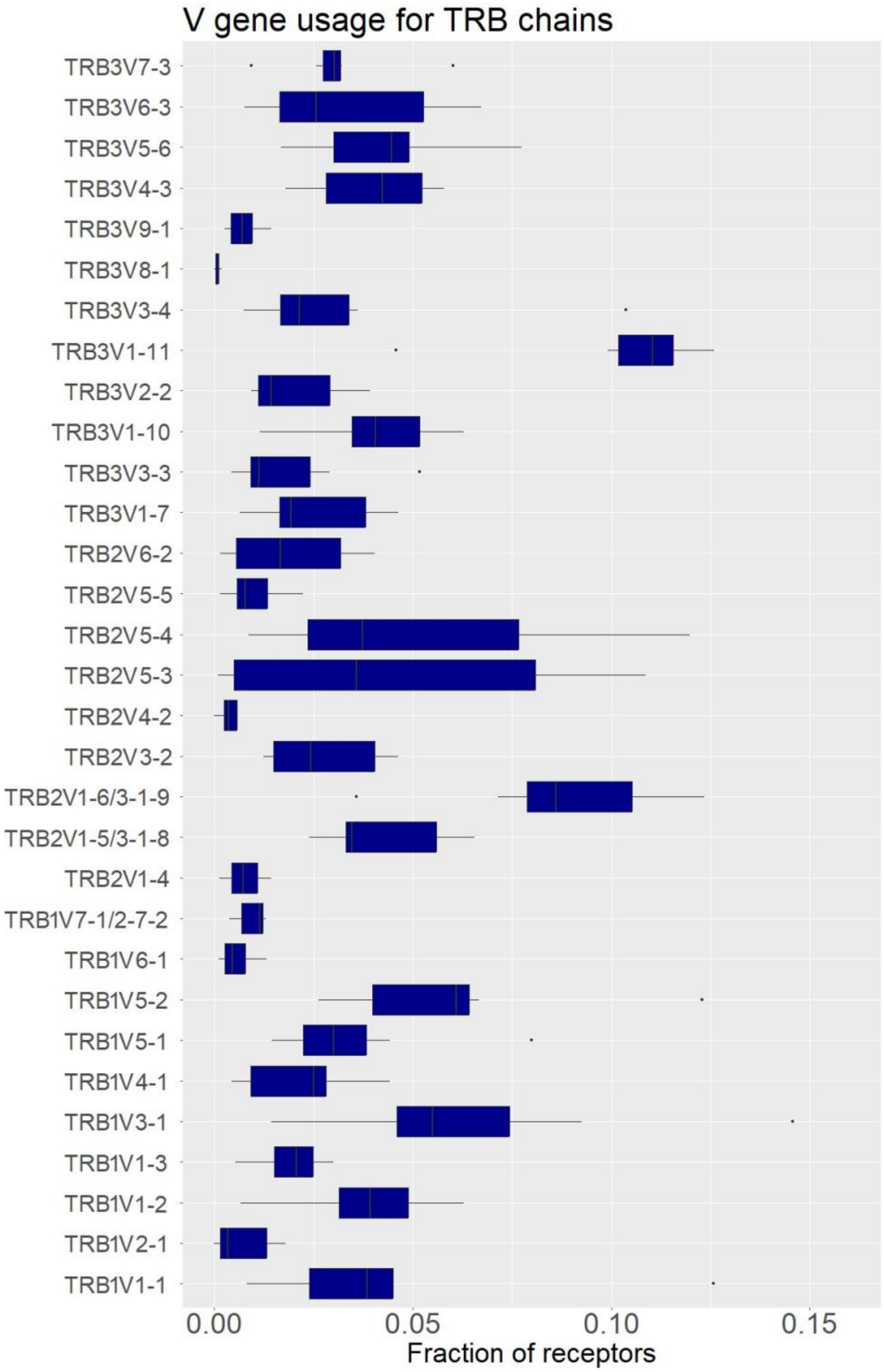

V gene usage for TR beta chains. Genes are ordered based on their subgroup name and individual gene name. One sample was used from each fish in the calculation (no technical replicate) of gene usage, error bars represent standard deviation between the different fish.

Supplementary figure 6

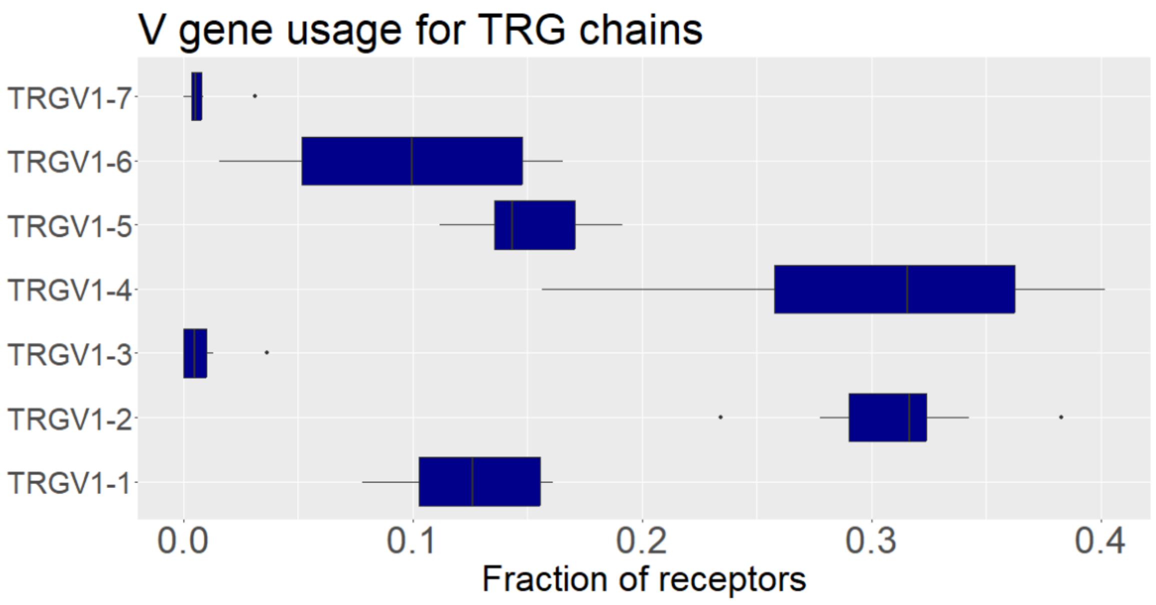

V gene usage for TR gamma chains. Genes are ordered based on their subgroup name and individual gene name. One sample was used from each fish in the calculation (no technical replicate) of gene usage, error bars represent standard deviation between the different fish.
